# Reconsidering antiviral prophylaxis in HBsAg-negative, anti–HBc-positive patients treated with rituximab

**DOI:** 10.1097/HC9.0000000000000903

**Published:** 2026-02-26

**Authors:** George Papatheodoridis, Jessica Hwang

**Affiliations:** 1 First Academic Department of Gastroenterology, Medical School, National and Kapodistrian University of Athens, General Hospital of Athens “Laiko,” Athens, Greece; 2 Department of General Internal Medicine, The University of Texas MD Anderson Cancer Center, Houston, Texas, USA

Hepatitis B virus (HBV) reactivation (HBVr) is a potentially life-threatening complication in patients undergoing immunosuppressive therapy. While the risk of HBVr is highest in patients with chronic HBV infection (HBsAg-positive), HBsAg-negative, anti–HBc-positive patients with presumed resolved HBV infection are also at risk for HBVr.^1^,^2^,^3^,^4^,^5^,^6^ The risk of HBVr is high among patients with hematologic malignancies and is particularly elevated among those treated with anticancer therapies containing rituximab,^1^,^2^,^3^,^4^,^5^,^6^ but the risk of HBVr is likely increased in patients receiving any B-cell depleting agent for any disease.

All clinical guidelines reflect growing awareness of the risk of HBVr in HBsAg-negative, anti–HBc-positive patients, although there are differences among the guidelines; some recommend antiviral prophylaxis but also acknowledge that careful monitoring is an alternative to antiviral prophylaxis if patients and providers can adhere to consistent follow-up.^1^,^2^,^3^,^4^,^5^ Despite the existence of these national clinical guidelines, practice patterns remain variable, and the evidence base guiding prophylaxis versus careful monitoring is limited.

In this issue of *Hepatology Communications*, Tabataba Vakili et al.^7^ present the results of a multicenter, randomized, placebo-controlled trial comparing the efficacy of 2 strategies in preventing clinically significant HBVr in HBsAg-negative, anti–HBc-positive patients undergoing rituximab-based anticancer therapy for lymphoma: (1) tenofovir alafenamide (TAF) and (2) a simple monitoring strategy based on only HBsAg and alanine aminotransferase (ALT) testing during standard oncology follow-up. The primary endpoint was HBsAg seroreversion, and a key secondary endpoint was HBVr, defined (as in most similar studies^6^,^8^) as HBsAg seroreversion and/or HBV DNA reappearance in patients with undetectable HBV DNA at baseline or an increase of >1 log_10_ IU/mL in patients with detectable HBV DNA at baseline. Patients were monitored every 3–4 weeks during chemotherapy, and every 6 weeks thereafter, while HBV DNA was measured post-hoc unless HBsAg seroreversion and/or a significant elevation of ALT occurred. The 2 strategies had similar efficacy: patients in the TAF and placebo arms had similar rates of HBsAg seroreversion (2 of 20 patients and 0 of 22 patients, respectively), reappearance or increase of HBV DNA [31 episodes in 10 of 20 patients (peak: 4260 IU/mL) and 29 episodes in 11 of 22 patients (peak: 3440 IU/mL), respectively], and high HBV DNA level (>1000 IU/mL) (3 of 20 patients and 3 of 22 patients, respectively), and no patient experienced a high ALT level [>2 times the upper limit of normal (×ULN)] or symptoms or signs of acute or chronic liver injury. Thus, the authors concluded that TAF can be safely used as prophylaxis in HBsAg-negative, anti–HBc-positive patients undergoing rituximab-based anticancer therapy for lymphoma, and that, alternatively, HBsAg and ALT testing without routine HBV DNA monitoring can effectively identify HBVr, prompting antiviral treatment and therefore may be an alternative to universal prophylaxis in well-monitored settings. The authors’ findings offer a timely and important contribution to this evolving field, challenging assumptions, current recommendations, and expert opinions^1^,^2^,^3^,^6^,^8^ and suggesting the need to reevaluate current strategies.

These interesting findings, however, should be interpreted with caution for several reasons. The study^7^ was terminated prematurely due to slow enrollment and thus had insufficient power for accurate assessment of differences between the groups in the main endpoint, HBsAg seroreversion. Only 42 patients were actually enrolled, far fewer than the planned 118 patients. Moreover, the authors’ prediction of a 15% HBsAg seroreversion rate in the placebo arm may have been high, as the pooled rate of HBVr based on HBV DNA monitoring seems to be 10%–11% in this setting.^9^

Another important limitation of this and other similar studies^6^,^8^ is the use of HBVr (by various definitions) as an endpoint, rather than the most clinically relevant endpoint, HBVr-associated hepatitis or severe hepatitis (ALT>10×ULN or baseline, ALT>3×ULN or baseline and bilirubin>2×ULN, or international normalized ratio >1.5),^8^ which may result in hepatic decompensation (ascites, hepatic encephalopathy, and/or variceal bleeding) or even death. There is no doubt that HBVr-associated severe hepatitis may occasionally occur in HBsAg-negative, anti–HBc-positive patients with lymphoma receiving rituximab-containing regimens without antiviral prophylaxis, especially if HBVr is not promptly recognized and treated. In contrast, HBVr has not been reported in such patients receiving antiviral prophylaxis, although publication bias may exist since failures of prophylaxis might not have been reported.^6^,^7^,^10^ Thus, a key question is: What is the risk of HBVr-associated severe hepatitis in patients under close monitoring and initiation of antiviral upon detection of HBVr (“upon indication”)? Unfortunately, this question may never be adequately addressed due to the need for very large sample sizes. Assuming that the risk of HBVr-associated severe hepatitis is 2% despite indication of antiviral therapy, 12,000 or 856 patients per arm would be required to reliably detect a reduction of this risk to 0% or 0.5%, respectively. Although no case of HBVr-associated severe hepatitis has been observed in other similar small studies,^6^,^8^ the 95% confidence interval for the 0% rate of severe HBVr in the control group in the Tabataba Vakili et al. study^7^ (n=22 patients) was wide: 0%–13.6%. Therefore, the core ethical and clinically relevant question is how great a risk of severe hepatitis clinicians and patients are willing to accept before deciding to initiate a preventive antiviral medication that is safe and well tolerated, keeping in mind that the number needed to treat to prevent one case of severe HBVr would be 50.

The Tabataba Vakili et al. study^7^ also raises important questions about the utility of prophylactic antiviral therapy in HBsAg-negative, anti–HBc-positive patients. Despite its rigorous design, the trial did not demonstrate a protective effect of TAF against HBsAg seroreversion and HBVr, which is in contrast with findings of previous studies assessing the efficacy of antiviral prophylaxis in this setting.^6^,^9^ Therefore, concerns about patients’ adherence to therapy were raised. In any case, this finding may invite clinicians and researchers to reconsider whether close monitoring might be a more appropriate and resource-conscious strategy than universal prophylaxis in certain patient populations. However, given that no case of HBVr-associated severe hepatitis has been reported in patients receiving antiviral prophylaxis, implementing a close monitoring approach instead of universal antiviral prophylaxis should not be undertaken without careful consideration, despite the potential adherence issues that might be more pronounced in real-life cohorts.^6^,^9^ What constitutes “close monitoring”? How frequently should ALT, HBsAg, and/or HBV DNA be assessed, and who is responsible for ensuring adherence to a monitoring schedule? In real-world settings, gaps in communication between hepatology, oncology, and primary care providers can lead to missed opportunities for early detection and intervention. Moreover, patients undergoing systemic anticancer therapy may face logistical and emotional barriers to consistent follow-up.

Based on current data, the time has not yet come to change the current recommendations for the management of HBsAg-negative, anti–HBc-positive patients receiving B-cell depleting therapy, but there is increased interest and space to learn more. Additional data is needed and is best suited to be sourced from collaborative initiatives. To start, a repository of patients treated with these agents to include the indication for therapy, duration, and dose of administration, other treatments received, and clinical laboratory test results, including ALT, HBsAg, and HBV DNA, would be useful. Ultimately, a large, multicenter and/or international clinical trial of HBsAg-negative, anti–HBc-positive patients receiving B-cell depleting therapies randomized to prophylaxis versus upon indication antiviral therapy with a primary endpoint of HBVr-associated hepatitis, along with clinically meaningful secondary endpoints, could generate robust data that would allow therapeutic interventions to be made with greater confidence. Beyond research, strengthening interdisciplinary collaboration will be essential. Oncology teams can work closely with hepatologists and primary care providers to develop personalized monitoring plans that are feasible, evidence-based, and clearly communicated. Electronic health record systems can play a role in facilitating shared care, but only if such systems are supported by institutional commitment and provider engagement. Ultimately, a coordinated approach will be key to ensuring that HBsAg-negative, anti–HBc-positive patients receive safe and effective care during immunosuppressive therapy.
